# Consequences of Cathodal Stimulation for Behavior: When Does It Help and When Does It Hurt Performance?

**DOI:** 10.1371/journal.pone.0084338

**Published:** 2014-01-07

**Authors:** Nazbanou Nozari, Kristina Woodard, Sharon L. Thompson-Schill

**Affiliations:** Department of Psychology, University of Pennsylvania, Philadelphia, Pennsylvania, United States of America; The Ohio State University, Center for Cognitive and Brain Sciences, Center for Cognitive and Behavioral Brain Imaging, United States of America

## Abstract

Cathodal Transcranial Direct Current Stimulation (C-tDCS) has been reported, across different studies, to facilitate or hinder performance, or simply to have no tangible effect on behavior. This discrepancy is most prominent when C-tDCS is used to alter a cognitive function, questioning the assumption that cathodal stimulation always compromises performance. In this study, we aimed to study the effect of two variables on performance in a simple cognitive task (letter Flanker), when C-tDCS was applied to the left prefrontal cortex (PFC): (1) the time of testing relative to stimulation (during or after), and (2) the nature of the cognitive activity during stimulation in case of post-tDCS testing. In three experiments, we had participants either perform the Flanker task during C-tDCS (Experiment 1), or after C-tDCS. When the Flanker task was administered after C-tDCS, we varied whether during stimulation subjects were engaged in activities that posed low (Experiment 2) or high (Experiment 3) demands on the PFC. Our findings show that the nature of the task during C-tDCS has a systematic influence on the outcome, while timing *per se* does not.

## Introduction

Transcranial Direct Current Stimulation (tDCS) is a non-invasive technique used to modulate behavior by applying electrical direct current to the scalp via two surface electrodes. Application of current alters the membrane potential of neurons, with anodal stimulation resulting in depolarization and cathodal stimulation in hyperpolarization [1]–[4]. Hence, anodal stimulation is considered to promote neuronal excitation in stimulated areas, whereas cathodal stimulation, neuronal inhibition. In behavioral terms, this usually translates into anodal tDCS causing improvement in performance, and cathodal tDCS causing a decline in performance (unless inhibitory neurons are inhibited, resulting in facilitation by cathodal stimulation). The literature is, in general, in support of this conclusion.

Evidence for behavioral facilitation of anodal stimulation includes – but is not limited to – improving associative verbal learning [5], language performance in picture naming [6], implicit motor learning [7], visuomotor performance [8], working memory [9], selective attention [10], and associative thought [11]. Examples of behavioral inhibition of cathodal stimulation include reduction in one's ability to filter out irrelevant information in categorization tasks [12], reduction of tactile perception [13], impairment of visual stimulus detection [14], poorer pitch matching performance [15], degradation of performance on cued shifts between global and local features [16], reduction of one's propensity to punish unfair behavior [17], and impaired working memory performance [18].

However, null effects of cathodal stimulation have also been reported on complex verbal associative thought [11], associative verbal learning [5], verbal fluency [19], picture naming [6], working memory [20], contrast sensitivity in the visual cortex [21], and probabilistic classification learning [22]. Moreover, some have reported cathodal stimulation to cause facilitation in picture naming in aphasic patients [23], in comprehension in subacute stroke patients [24], and in deception in an interrogation following a thief role-play [25]. Although the facilitatory effect of cathodal stimulation in individuals with brain damage is often ascribed to successful suppression of abnormal cortical activity [24], this interpretation, even if correct, does not explain the variety of results obtained with cathodal stimulation. A recent meta-analysis by Jacobson, Koslowsky, and Lavidor [26] highlights the degree of this variability: Fairly consistent results have been found for both anodal (facilitation in 14 out of 18 cases; 78%) and cathodal (inhibition in 13 out of 15 studies; 87%) stimulation when motor effects are studied, but there is considerably much less consistency in stimulation effects in cognitive tasks. Facilitation in behavior has been reported in 26 out of 32 (81%) of studies using anodal stimulation, but inhibition has been reported in only 10 out of 21 (48%) of studies employing cathodal stimulation.

The variability in behavioral reports of cathodal effects is cause for worry, because in some cases results are compatible with more than one interpretation, depending on what cathodal stimulation is assumed to be doing. For example, Chrysikou et al. [27] had participants generate either an object's common use (e.g., bat: to hit a baseball) or uncommon use (e.g., bat: to smash a window) under concurrent cathodal stimulation of the left prefrontal cortex (PFC). They found that cathodal stimulation improved the generation of uncommon, but not common, uses of objects. If cathodal stimulation is assumed to have an inhibitory effect on the PFC (as assumed by the authors), then the results can be interpreted as limiting the top-down influence of the PFC, and allowing bottom-up perceptual influences to guide decisions rather than rule-based thought. However, if one allows for the possibility that cathodal stimulation may have caused excitation of PFC neurons, the results could alternatively be explained as the consequence of facilitating subjects' ability to override a prepotent response in favor of less potent options [28], [29].

In the present study, we set out to identify some of the causes of variability in reports of cathodal stimulation. This variability can have two sources: the effect of the direct current on biological tissue, and the effect on the behavioral patterns that emerge from changes at the neuronal level. While the two are obviously related, we believe that exploring either by itself has merit, as changes in behavior are not necessarily predictable by changes at the neuronal level. Moreover, if there are overt changes in behavior, there is good motivation to further investigate the underlying changes in the biological tissue mediating that behavior. As such, we chose to explore stimulation-induced changes in behavior in this paper, hoping that the result would provide directions for more detailed electrophysiological studies in the future.

In what follows, we briefly discuss the factors that lend themselves less to experimental control, and then move on to discussing two variables that we have reason to believe might matter in changing the behavioral outcome of cathodal stimulation. We then present three experiments, along with a control experiment, in which we systematically manipulate these two variables and test their influence on the stimulation outcome, as subjects perform a simple task (a Flanker task), held constant across all experiments.

### Factors that may change the behavioral outcome of stimulation

The diversity of cathodal findings could result from individual differences in response to tDCS [30]–[32], individual differences in the laterality of cognitive functions, or variations in tDCS practices. Subject-related factors are less within the power of experimenter to control. Responsiveness to tDCS is very difficult to estimate *a priori*, and excluding subjects after participation induces undesirable biases. As for the laterality of functions, control of handedness is a useful, but not a perfect, control. Given the difficulty in controlling these factors, it is most important to reduce uncertainty by controlling the factors involved in the experimental design. One such critical factor is the montage. Variations in montage and placement of the reference electrode have been discussed extensively elsewhere [2], [33]–[40]. Other design variables, however, have received little attention. In this paper, we focus on two such factors that are likely candidates for changing stimulation effects on behavior, given the past literature: (1) the temporal relationship between the cognitive test and the stimulation, and (2) the nature of the task performed during the stimulation.

A survey of the cathodal cognitive studies that have yielded different results implicates timing as a possible contributing factor. For example, Lupyan et al. [12] reported a decline in performance when the cognitive task was performed almost entirely during tDCS. Monti et al. [23], on the other hand, found behavioral facilitation when the cognitive task was applied post-tDCS (although the authors interpret this effect as inhibition of inhibition). Finally, Flöel et al. [5] found null effects on behavior when part of the task was completed during tDCS, and part of it, post-tDCS. Moreover, within-study changes of cathodal effects have also been observed as a function of time. Stone and Tesche [16] stimulated the parietal lobe during a task in which participants were cued to shift attention between global features (the letter formed by a group of letters) and local features (the individual letters). During tDCS, cathodal stimulation was found to impair performance on all cued shifts between global and local features. However, post-tDCS, cathodal subjects did not show impairment (null effect).

The possible effect of timing on the direction of stimulation effects is also suggested by the electrophysiological data in motor learning. tDCS has been proposed to influence neuronal activity via two mechanisms: *gating*, which refers to transiently increasing the excitability of the motor cortex via stimulation concurrent with the task, and *homeostatic plasticity*, which refers to lowering the threshold for induction of synaptic plasticity by decreasing neuronal activity before the task [41]. While gating usually has a straight forward effect (e.g. anodal tDCS leading to excitatory effects), homeostatic plasticity may induce in the task following stimulation, an effect opposite of the one observed at the time of stimulation. For example, Lang, Nitsche, Sommer, and Tergau [42] showed that anodal tDCS of the motor cortex – which induced excitability changes online – induced impaired performance after the end of the stimulation. These effects, however, have not been investigated in cognitive tasks, and their behavioral outcome is uncertain.

Furthermore, timing alone may not fully explain the diversity of reports. Both Friederici et al. [43] and Cerruti and Schlaug [11] examined the effect of cathodal stimulation of the left PFC on a language task post-tDCS, and obtained different results. In Cerruti and Schlaug's [11] experiment, participants spent 16 minutes receiving stimulation with no task, and completed a verbal fluency task during the last 4 minutes of stimulation. After the 20-minute stimulation period was over, they were given a Remote Association Task (RAT). On each trial they saw a set of words and were asked to generate a word that best united the set. The authors failed to find an effect of cathodal stimulation on performance in the RAT. Friederici et al. [43] trained participants in the artificial grammar of a novel language during the stimulation phase. While they did not find overt behavioral differences between cathodal and sham stimulation in a grammatical judgment task administered post-tDCS, they showed different event-related brain potentials (ERPs) in this phase. Although comparing changes to overt behavior and to evoked potentials is less than ideal, the difference between the results of these two studies, both of which looked for post-tDCS effects, brings up the possibility that what participants do under stimulation might have a direct effect on stimulation results. If this is true, tDCS must be viewed as a highly dynamic technique, the effects of which depend critically on the activity of the cortical region at the time of stimulation.

In this paper, we test the effects of timing (post vs. during) and task variation (whether the subjects' cognitive activity at the time of tDCS administration employs similar operations as the test task or not) when cathodal tDCS is applied to the left PFC. We chose the letter Flanker task [44] as the test task for all the experiments for the following reasons: (a) The task is simple, easy to learn, and has clear indices for better/worse performance, in accuracy and reaction times (RTs), minimizing the possibility for alternative interpretations of results. (b) The simple nature of the task allows for many trials (≈700) to be accommodated within the 20-minute period of stimulation, so that a comparison can be made between the results during and post tDCS, while obtaining reliable estimations of performance at the individual level. And (c) the task poses demands on the PFC, both on working memory and on selective attention (see Methods for more details), in both cases, the effect goes in the same direction, at least when anodal stimulation has been tried. Previous studies have shown that anodal stimulation enhances performance on working memory [20], [45]–[47] and selective attention on the central letters (10), typically presumed to be the consequence of neural excitation. Importantly, we keep the task constant across all experiments. Thus, even without making assumptions about the relationship between neural and behavioral responses, a change in performance from one experiment to another would indicate a different effect.

In Experiment 1, participants completed the Flanker task during concurrent cathodal stimulation. By the virtue of the design, no additional filler activity was needed during stimulation. If Cathodal tDCS has inhibitory effects, it is reasonable to expect them here, where the task has full overlap with tDCS and engages the neural tissue targeted by the stimulation. In Experiments 2 and 3 we administered the Flanker task post-tDCS. If timing is the main factor in reversing/altering cathodal effects, we would expect Experiments 2 and 3 to show a similar pattern to each other, and one that would be different from the pattern observed in Experiment 1. Crucially, we varied what subjects were doing *during* stimulation between Experiments 2 and 3. In Experiment 2, subjects were engaged in an activity that posed minimal executive demands: They performed a simple categorization procedure. A control experiment followed this experiment to rule out effects of the reference electrode placement. In Experiment 3, on the other hand, we increased executive demands placed on subjects during stimulation: On each trial, participants saw the picture of one object (e.g. ‘bag’) or another (e.g. ‘ball’) and after naming each picture several times in random order, were told they now had to name the other object upon viewing each object (i.e. say ‘bag’ when you see a ‘ball’). Overriding of the dominant response in this task requires strong cognitive control, which engages the left PFC. If it is not timing *per se*, but the nature of the activity during stimulation, that determines the outcome of stimulation, then we would expect a different pattern of results between Experiments 2 and 3. Specifically, we would expect that the results of Experiment 3 would pattern similarly with those of Experiment 1, because in both experiments PFC was engaged at the time of cathodal stimulation. However, Experiment 2 might be expected to show a different pattern of results, because in this case the task during stimulation does not strongly engage the area being stimulated.

## Methods

### Ethics Statement

This research was approved by the IRB of the University of Pennsylvania. All participants signed HIPPA forms, and gave written consent for tDCS administration, prior to participation, in accordance with the IRB-approved experimental protocol.

In what follows, we first describe the letter Flanker task, our stimulation parameters and the analysis method that are common to multiple experiments and then move on to describing each experiment individually.

### Experimental task (letter Flanker)

Experimental materials consisted of four capital letters (X, S, H, P) appearing in arrays of five in the center of the screen in size 18 Courier New presented to participants at a distance of 22 inches from the monitor. E-prime 2.0 (PST softwares, Pittsburgh, PA, USA) was used to present the stimuli. A response was required to the central letter, ignoring the four flanking letters. Participants were instructed to press one key if the target letter was an X or an S, and another key if the target was a P or an H. They used their right index and middle fingers to respond, and the assignment of response keys to letters was counterbalanced across participants. 50% of the trials were *congruent* (C) trials where all letters were the same (e.g. SSSSS). In the remaining 50% of trials the central letter was different from the flankers. In half of these trials, the response to the central letter was the same as the response to the flankers (e.g. SSXSS). In the other half, the *incongruent* (I) condition, a different response was required to the central stimulus (e.g. SSPSS). The stimuli were presented for 800 ms (decided based on piloting before the study) during which participants made their response. Trials were presented in random order and the inter-trial interval (during which a fixation cross appeared in the center of the screen) was jittered between 500 ms and 1500 ms, with each interval determined by a random number chosen from a uniform distribution. The design parameters were chosen to maximize demands on the PFC. There are two types of demands posed by the letter Flanker task: (1) Inhibition of the flankers and attending to the central letter (in the incongruent trials), and (2) keeping in mind four stimulus-response mapping options (working memory; in all trials). In addition, having a short deadline and unpredictable inter-trial interval calls for stronger cognitive control. Participants received 60 practice trials (excluded from the analyses), followed by two blocks of 352 trials each. The entire task, including the practice phase, took about 20 minutes.

### Direct Current Stimulation

Saline-soaked sponge electrodes with the surface area of 25 cm^2^ were used to deliver continuous direct current generated by a battery-driven continuous current stimulator (Magstim Eldith 1 Channel DC Stimulator Plus, Magstim Company Ltd., Whitland, Wales). During stimulation, 1.5 mA current (with a 30 seconds ramp up and ramp down) was applied for 20 minutes. Stimulation lasted only 30 seconds during sham. The cathode was placed over the F7, according to the 10–20 international system for EEG electrode placement. Unless otherwise specified, the anode was placed over the right mastoid. This montage was chosen because among the few studies in which cathodal stimulation was used to target cognitive control, it has been a common montage [12], [27], [45], and covers parts of both ventrolateral and dorsolateral PFC.

### Data analysis

All experiments use a between-subject design. As the dependent variable for accuracy is categorical (and binomially distributed), we chose a logistic model to analyze the data. This type of model avoids the violation of homoscedasticity, which is one of the main assumptions of ANOVA, when the proportion of correct responses is close to zero or one. The models used are generalized linear multi-level mixed models with the random effect of subjects, to stay as close to the traditional ANOVA in treating subjects as a random factor, while at the same type avoiding the violation of models' assumptions. To be consistent throughout, we analyzed RT's with comparable models.

In the Flanker task, the fixed effects included condition(C vs. I), stimulation (cathodal vs. sham) and the interaction between the two. The random intercept for subjects corrects for subjects' variability in overall task performance. When accuracy was analyzed, a logistic version of the model is used, in which each response received a binary code of 0 for error and 1 for correct. For analyzing RT, a lower cutoff of 250 ms was imposed, and RTs shorter than this were discarded, because they were very unlikely to reflect real decision processes. 4%, 4%, and 6% of responses were discarded from Experiments 1–3 respectively, because they met this criterion. Given the 800 ms deadline for responding, no upper-limit cutoff was deemed necessary. Only the RTs from correct trials were entered in the analysis. Trials where different letters mapped onto the same response (i.e. SSXSS) were not included in the analyses, because it is impossible to determine whether participants in fact succeeded or failed at inhibiting the flankers (See Appendix S1 for the details of accuracy and RT's for these trials). The C and I trials used in the analyses, thus, correspond to the standard Flanker task with two canonical conditions.

## Experiment 1

This Experiment was an attempt to replicate previous cathodal experiments where the experimental task was completed during stimulation. By the virtue of this, no filler activity was needed.

### Participants

Sixteen (8 cathodal, 8 sham; 9 women) right-handed, native speakers of English between the ages of 18 and 26 participated in the experiment for cash. No participants were pregnant, took psychotropic/anti-convulsive drugs, or had a history of strokes, seizures, or head trauma.

### Procedure

Instructions were given first, and practice (≈2 min) started simultaneously with the stimulation. Once practice was over, participants moved on to the main task and completed the entire task during cathodal stimulation.

### Results and Discussion

**Figure 1:**
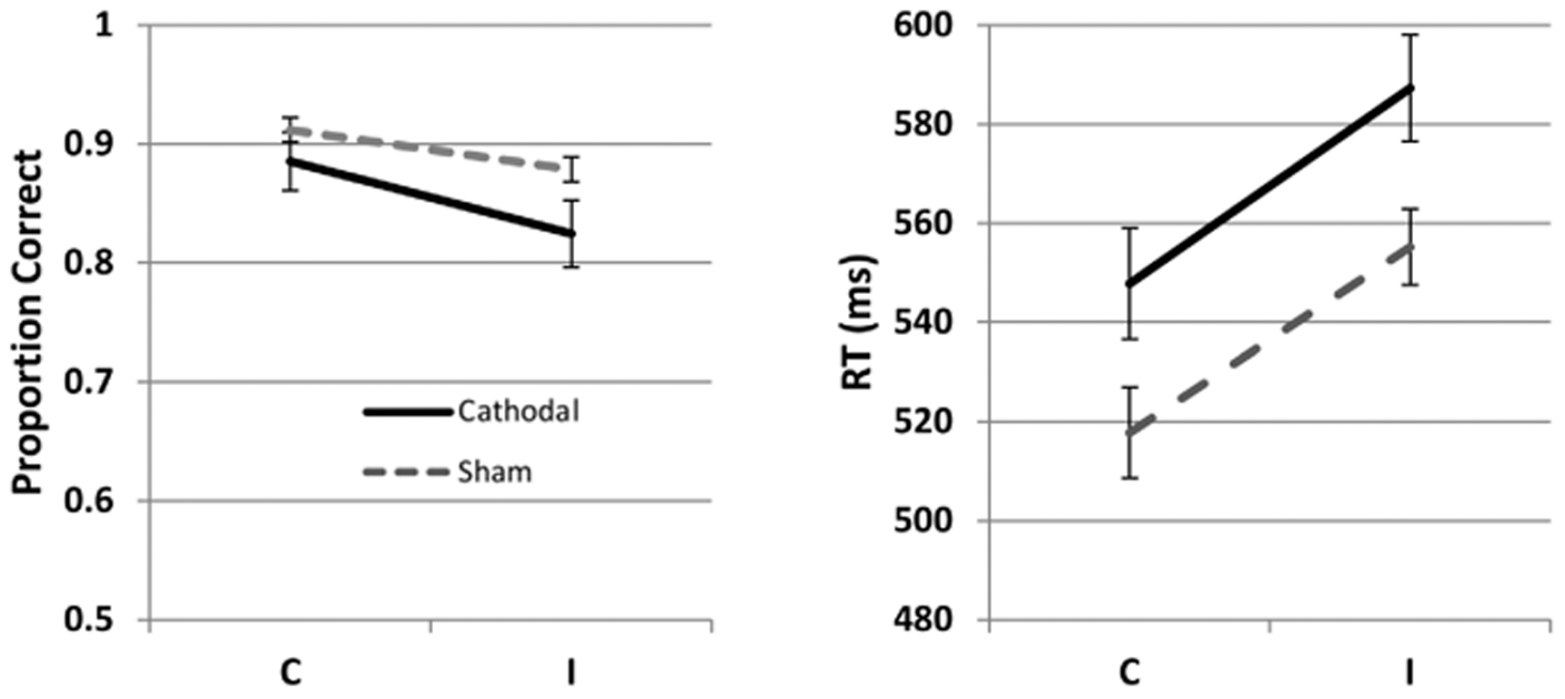
Mean accuracy and reaction times (± SE) in the Flanker task in Experiment 1. The left panel shows mean accuracy, and the right panel shows reaction times. C  =  Congruent; I  =  Incongruent.

Mean accuracy and RT's for the cathodal and sham groups are presented in Tables 1 and 2, and summarized in Fig. 1. The results of the multilevel model analysis are presented in Table 3. For both accuracy and RTs, there was a main effect of Condition, such that performance was better (more accurate and faster) in the C, compared to the I, condition (z = −5.51, p<.001 for accuracy, and t = 12.61, p<.001 for RT's). While the accuracy of performance was not significantly different between the two groups (z = 0.91, p = 0.37), the cathodal group was reliably slower (t = −2.22, p = 0.027) than sham. The interaction between Stimulation and Condition (C vs. I) did not reach significance (z = 1.09, p = 0.28 for the accuracy analysis, and t = −0.62, p = .53 for the RT analysis).

**Table 1:** Mean accuracy (±SE) for the Congruent and Incongruent conditions in cathodal and sham for experiments 1–3.

	Cathodal				Sham			
	Congruent		Incongruent		Congruent		Incongruent	
	Mean	SE	Mean	SE	Mean	SE	Mean	SE
**Exp 1**	0.89	0.025	0.82	0.028	0.91	0.010	0.88	0.10
**Exp 2a**	0.92	0.013	0.87	0.018	0.86	0.021	0.77	0.027
**Exp 2b**	0.88	0.018	0.83	0.020	0.86	0.021	0.77	0.027
**Exp 3**	0.89	0.020	0.82	0.028	0.88	0.016	0.79	0.026

**Table 2:** Mean reaction time (±SE) for the Congruent and Incongruent conditions in cathodal and sham for experiments 1–3.

	Cathodal				Sham			
	Congruent		Incongruent		Congruent		Incongruent	
	Mean	SE	Mean	SE	Mean	SE	Mean	SE
**Exp 1**	547.8	11.2	587.3	10.7	517.8	9.17	555.2	7.68
**Exp 2a**	509.8	14.2	558.7	14.7	534.1	11.5	582.2	10.9
**Exp 2b**	518.4	9.26	560.3	9.74	534.1	11.5	582.2	10.9
**Exp 3**	537.8	11.8	581.7	11.03	520.1	11.2	556.4	10.3

**Table 3:** Summary of the fixed and random effects in the multi-level model in Experiment 1.

Accuracy					
	Fixed Effects	Coefficient	SE	z	Pr(>|z|)
	Intercept	2.15573	0.16255	13.262	<0.001
	Condition	−0.51472	0.09344	−5.508	<0.001
	Stimulation	0.2092	0.23118	0.905	0.365
	Condition*Stimulation	0.15387	0.14094	1.092	0.275
	**Random Effects**	**Variance**			
	Subject intercept	0.18001			

RT					
	Fixed Effects	Coefficient	SE	t	Pr(>|t|)
	Intercept	548.788	9.746	56.31	<0.001
	Condition	39.539	3.135	12.61	<0.001
	Stimulation	−30.528	13.779	−2.22	0.0267
	Condition*Stimulation	−2.723	4.377	−0.62	0.5338
	**Random Effects**	**Variance**			
	Subject intercept	735.63			

Stimulation has two levels (cathodal and sham), and Condition has two levels (C and I). See text for details. *signifies an interaction.

Together, these results point to an inhibitory effect of cathodal stimulation. Given that the effect of stimulation was found on both congruent and incongruent trials, but not on the difference between them, we suspect that stimulation affected the stimulus-response mapping aspect of the Flanker task.

## Experiment 2a,b

In Experiment 2 we aimed to test whether the timing of the experimental task in relation to stimulation matters. We, therefore, changed the timing of the letter Flanker task from during to after tDCS. During stimulation, participants did not engage in the Flanker task, or another activity requiring a comparable level of cognitive control as the Flanker task. Instead, they made a simple categorization judgment described below. We could have had subjects do nothing during stimulation, as has been done in some studies, but to ensure that all subjects were equally engaged, we decided on an activity. To make sure that the results of Experiment 2a were not an artifact of the placement of the reference electrode, a control group (2b) were run with a different placement of the reference electrode.

### Montage

Group 2a was run under the canonical montage (F7- right mastoid). Group 2b was run with cathode on F7, but the reference electrode was moved to the right supraorbital area. Sham subjects received one or the other montage and were used as a baseline of comparison for groups 2a and 2b.

### Participants

Across both experiments, 31 (9 cathodal with the reference electrode on the right mastoid, 8 cathodal with the reference electrode on the right supraorbital, 14 sham; 18 women) individuals between ages of 18 and 34, meeting similar inclusion and exclusion criteria as Experiment 1 participated for cash.

### Procedure

Participants received cathodal stimulation of the left PFC for 20 minutes during which they were asked to judge whether a pictured animal, from one of three categories, had a fictitious property based on an explicitly instructed rule mapping the property to one category [46]. We chose this activity for reasons unrelated to the present study, but critically, it is not hypothesized to recruit PFC. Subjects made these categorization judgments for approximately 15 minutes, beginning 3 minutes after stimulation. Immediately after stimulation, subjects completed the Flanker task for the next 20 minutes.

### Results and Discussion

**Figure 2:**
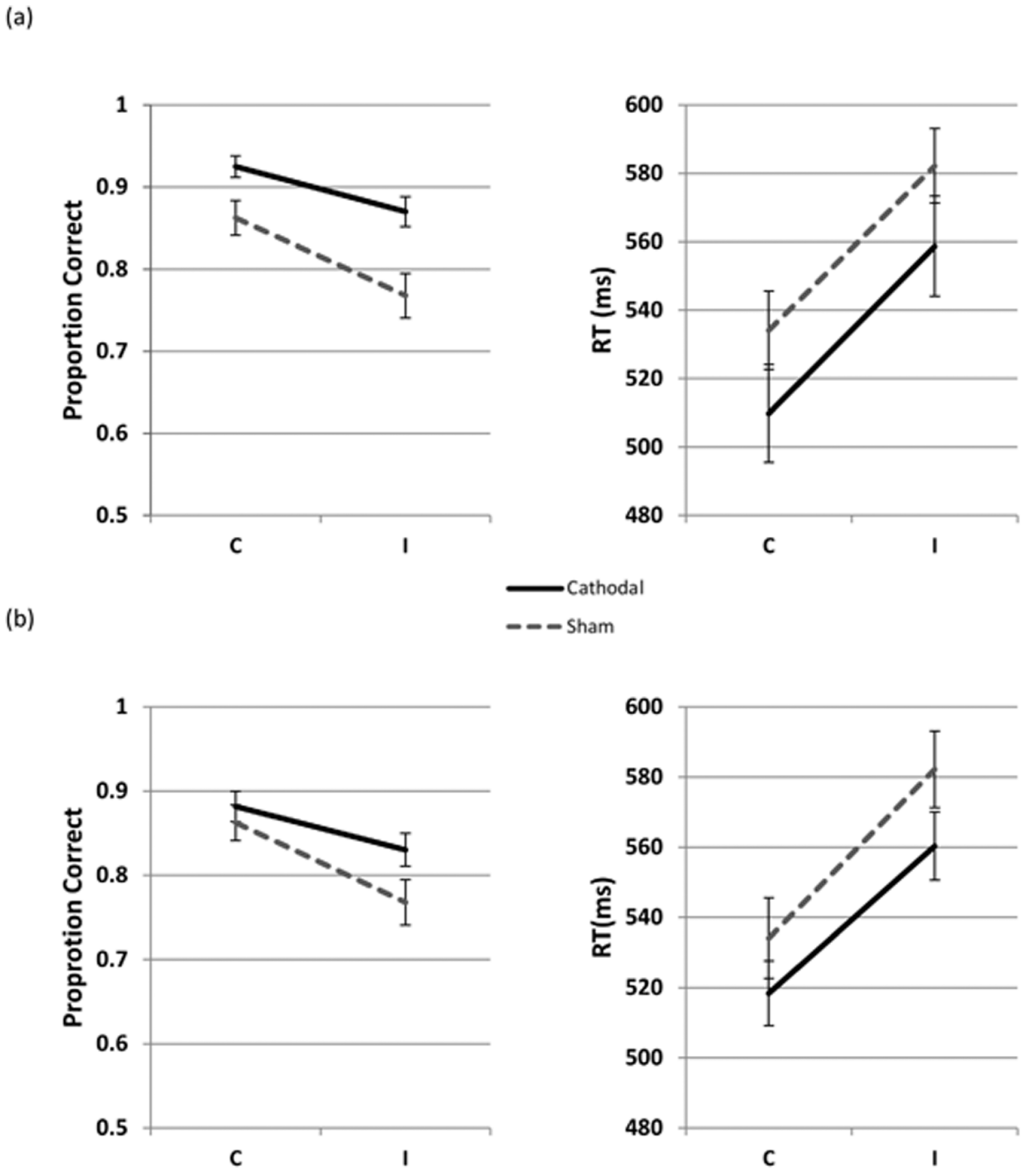
Mean accuracy and reaction times (± SE) in the Flanker task in Experiment 2. (a) Data from Experiment 2a (post-tDCS; right mastoid as the reference electrode site). (b) Data from Experiment 2b (post-tDCS; right supraorbital area as the reference electrode site). In both experiments stimulation was applied during an activity requiring low cognitive control. C  =  Congruent; I  =  Incongruent.

Figure 2a shows the accuracy and RTs in the Flanker task for the cathodal group with the canonical montage (i.e. reference electrode on the right mastoid) against sham (see also Tables 1 and 2). This cathodal group was significantly more accurate (z = −2.43, p = .015) and no slower than the sham group (t = 1.46, p = .14). The interaction between Stimulation and Condition (C vs. I) did not reach significance for either accuracy (z = −0.40, p = .68) or the RT analysis (t = −0.01, p = .99). Similar to Experiment 1, congruent trials were processed faster (t = 16.66, p<.001) and more accurately (z = −6.12, p<.001) than incongruent trials (Table 4).

**Table 4:** Summary of the fixed and random effects in the multi-level model in Experiment 2a.

Accuracy					
	Fixed Effects	Coefficient	SE	z	Pr(>|z|)
	Intercept	2.61753	0.2105	12.435	<0.001
	Condition	−0.62162	0.10156	−6.121	<0.001
	Stimulation	−0.65049	0.26747	−2.432	0.015
	Condition*Stimulation	−0.04824	0.12042	−0.401	0.689
	**Random Effects**	**Variance**			
	Subject intercept	0.35317			

RT					
	Fixed Effects	Coefficient	SE	t	Pr(>|t|)
	Intercept	510.33382	13.77973	37.04	<0.001
	Condition	49.18358	2.95177	16.66	<0.001
	Stimulation	25.84498	17.66598	1.46	0.1435
	Condition*Stimulation	−0.04269	3.86767	−0.01	0.9912
	**Random Effects**	**Variance**			
	Subject intercept	1686.5			

Stimulation has two levels (cathodal and sham), and Condition has two levels (C and I). See text for details. * signifies an interaction.

In summary, more accurate responses under stimulation points to a clear facilitatory effect of cathodal tDCS in this experiment, quite the opposite of what we found in Experiment 1. To demonstrate the reliability of this difference, we built a model with accuracy as the dependent variable, and Stimulation (cathodal vs. sham), Condition (C vs. I), Experiment (Experiment 1 vs. Experiment 2a), and their interaction terms as independent variables. Table 5 presents the results of this analysis. In this model, the two-way interaction between Stimulation and Experiment was significant (z = −2.32, p = .021), showing that cathodal stimulation had a reliably different effect in Experiment 1 compared to Experiment 2. The three-way interaction between Stimulation, Condition and Experiment was not significant (z = −1.09, p = .27). In the same model with the RT as the dependent variable, the two-way interaction between Stimulation and Experiment was again significant (t = 2.35, p = 0.019), but the three-way interaction was not (t = 0.46, p = 0.65; see Table 5 for all other effects). In summary, the joint analysis of Experiments 1 and 2a point to clear differences of cathodal stimulation in these two experiments.

**Table 5:** Summary of the fixed and random effects in the multi-level model testing the effect in Experiment 1 vs. Experiment 2a.

Accuracy				
Fixed Effects	Coefficient	SE	z	Pr(>|z|)
Intercept	2.1631	0.19703	10.978	<0.001
Condition	−0.51583	0.09355	−5.514	<0.001
Stimulation	0.20384	0.27966	0.729	0.4661
Experiment	0.44987	0.27356	1.644	0.1001
Condition*Stimulation	0.15486	0.14102	1.098	0.2722
Condition*Experiment	−0.10541	0.13806	−0.764	0.4451
Stimulation*Experiment	−0.85399	0.36895	−2.315	0.0206
Condition*Stimulation*Experiment	−0.20286	0.18541	−1.094	0.2739
**Random Effects**	**Variance**			
Subject intercept	0.27884			

RT				
Fixed Effects	Coefficient	SE	t	Pr(>|t|)
Intercept	548.798	12.893	42.57	<0.001
Condition	39.539	3.179	12.44	<0.001
Stimulation	−30.535	18.23	−1.67	0.094
Experiment	−38.466	17.716	−2.17	0.0299
Condition*Stimulation	−2.729	4.438	−0.61	0.5387
Condition*Experiment	9.644	4.319	2.23	0.0256
Stimulation*Experiment	56.375	23.979	2.35	0.0187
Condition*Stimulation*Experiment	2.684	5.863	0.46	0.6471
**Random Effects**	**Variance**			
Subject intercept	1306.1			

Stimulation has two levels (cathodal and sham), Condition has two levels (C and I), and Experiment has two levels (Exp 1 and Exp 2a). See text for details. * signifies an interaction.

Given the typical claims made about the consequences of cathodal stimulation (namely, that is has inhibitory effects on behavior), we set out to replicate our results using a new group of participants and a slightly different montage, to test the possibility that facilitatory effects may arise from excitation of the visual cortex under the reference electrode. In Experiment 2b we moved the reference electrode from the right mastoid to the right supraorbital area. Figure 2b shows the accuracy and RTs for the cathodal group with the alternative montage (F7-right supraorbital) against sham (see also Tables 1 and 2). The facilitatory nature of the results did not change. This time there was evidence for facilitation on inhibition of the flankers (Table 6): there were reliably fewer errors in the I condition compared to C in cathodal compared to sham (z = −2.12, p = .034). Also, participants were marginally faster in the I, compared to the C, condition in the cathodal, compared to the sham, group (t = 1.83, p = 0.067). Similar to the previous experiments, there was a consistent benefit for the C trials in accuracy (z = −4.63, p<.001) and RT (t = 12.76, p<.001).

**Table 6:** Summary of the fixed and random effects in the multi-level model in Experiment 2b.

Accuracy					
	Fixed Effects	Coefficient	SE	z	Pr(>|z|)
	Intercept	2.06815	0.20503	10.087	<0.001
	Condition	−0.42977	0.09274	−4.634	<0.001
	Stimulation	−0.10346	0.25687	−0.403	0.687
	Condition*Stimulation	−0.23974	0.11307	−2.12	0.034
	**Random Effects**	**Variance**			
	Subject intercept	0.30733			

RT					
	Fixed Effects	Coefficient	SE	t	Pr(>|t|)
	Intercept	518.63	12.883	40.26	<0.001
	Condition	41.59	3.26	12.76	<0.001
	Stimulation	17.541	16.151	1.09	0.2775
	Condition*Stimulation	7.548	4.134	1.83	0.0679
	**Random Effects**	**Variance**			
	Subject intercept	1301.7			

Stimulation has two levels (cathodal and sham), and Condition has two levels (C and I). See text for details. * signifies an interaction.

In order to demonstrate the reliability of the difference between Experiment 1 and Experiment 2b, we built a model with accuracy as the dependent variable, and Stimulation (cathodal vs. sham), Condition (C vs. I), Experiment (Experiment 1 vs. Experiment 2b), and their interaction terms as independent variables. Table 7 presents the results of this analysis. When compared to Experiment 1, the three-way interaction between stimulation, condition and experiment was significant (z = −2.18,  = .029), showing that Cathodal tDCS in Experiment 2b significantly decreased the number of errors in the incongruent – vs. the congruent condition – compared to Experiment 1. Also, the two-way interaction between stimulation and experiment was significant for the model with RT as the independent variable (z = 2.18, p = .030), demonstrating that cathodal tDCS in Experiment 2b had a reliably different effect on RT's in both C and I conditions compared to Experiment 1 (See Table 7 for other effects).

**Table 7:** Summary of the fixed and random effects in the multi-level model testing the effect in Experiment 1 vs. Experiment 2b.

Accuracy				
Fixed Effects	Coefficient	SE	z	Pr(>|z|)
Intercept	2.16163	0.18837	11.475	<0.001
Condition	−0.51564	0.09353	−5.513	<0.001
Stimulation	0.20495	0.26747	0.766	0.4435
Experiment	−0.09481	0.26575	−0.357	0.7213
Condition*Stimulation	0.15468	0.141	1.097	0.2727
Condition*Experiment	0.08602	0.1317	0.653	0.5136
Stimulation*Experiment	−0.31089	0.35591	−0.874	0.3824
Condition*Stimulation*Experiment	−0.394	0.18072	−2.18	0.0292
**Random Effects**	**Variance**			
Subject intercept	0.25221			

RT				
Fixed Effects	Coefficient	SE	t	Pr(>|t|)
Intercept	548.795	11.69	46.95	<0.001
Condition	39.539	3.209	12.32	<0.001
Stimulation	−30.533	16.528	−1.85	0.0647
Experiment	−30.166	16.532	−1.82	0.0681
Condition*Stimulation	−2.727	4.48	−0.61	0.5427
Condition*Experiment	2.051	4.536	0.45	0.6511
Stimulation*Experiment	48.068	22.09	2.18	0.0296
Condition*Stimulation*Experiment	10.272	6.049	1.7	0.0895
**Random Effects**	**Variance**			
Subject intercept	1068.5			

Stimulation has two levels (cathodal and sham), Condition has two levels (C and I), and Experiment has two levels (Exp 1 and Exp 2b). See text for details. * signifies an interaction.

Together, Experiments 2a and 2b confirmed that cathodal effects can have true facilitatory effects on behavior, even in neurologically-healthy individuals, where abnormal cortical activity (the suppression of which has been taken to be the origin of facilitatory effects in cathodal experiments with brain-damaged individuals) is unlikely.

## Experiment 3

Cathodal stimulation in Experiment 1 caused a decline in performance, while Experiments 2a and 2b showed facilitatory effects of cathodal stimulation. These Experiments differed in two ways: temporal overlap of the Flanker task and stimulation, and the nature of subjects' cognitive activity during stimulation. To tease apart the influence of these two factors we conducted a final experiment, in which the timing of the Flanker task relative to stimulation was the same as in Experiment 2, but the activity in which subjects were engaged during the stimulation posed strong demands for cognitive control, hence engaging the cortical areas under stimulation.

### Participants

Twenty-two (12 cathodal, 10 sham; 13 women) individuals between ages of 18 and 25, meeting similar inclusion and exclusion criteria as Experiment 1 participated for cash.

### Procedure

Participants received cathodal stimulation of the left PFC for 20 minutes, during which they swapped the name of two objects. Initially, one of the two pictures appeared in random order and participants named them normally. After a few such trials, subjects were instructed to name picture B whenever picture A appeared and vice versa, as quickly as possible. Calling familiar objects by the wrong name is quite difficult and poses high demands on PFC-mediated cognitive control functions. This procedure began 3 minutes after the stimulation began, and took approximately 15 minutes to complete. After the 20-minute stimulation period was over, participants completed the Flanker task.

### Results and Discussion

**Figure 3:**
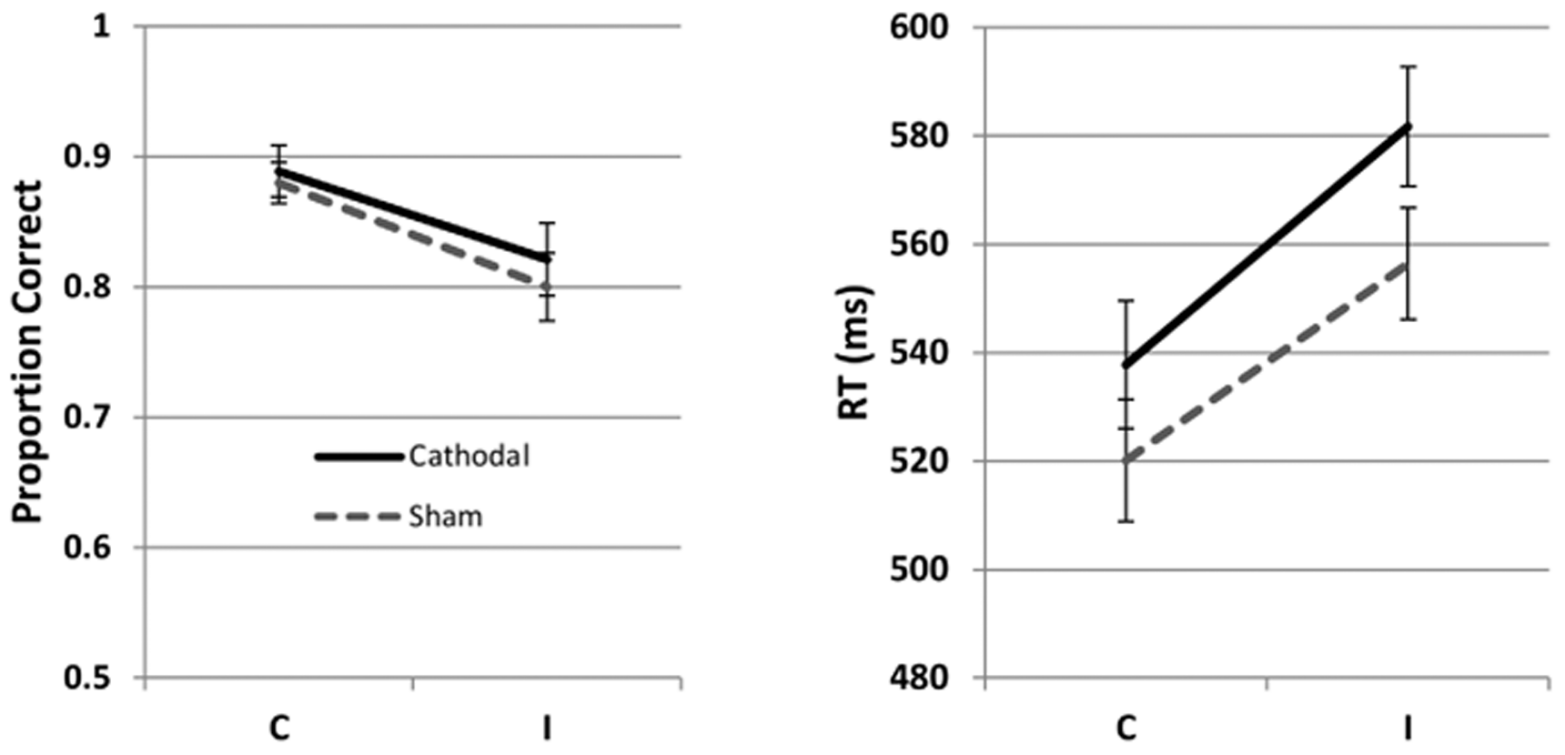
Mean accuracy and reaction times (± SE) in the Flanker task in Experiment 3. The left panel shows mean accuracy, and the right panel shows reaction times. Stimulation was applied during an activity requiring high cognitive control. C  =  Congruent; I  =  Incongruent.

Figure 3 shows the accuracy and RTs for the Flanker task administered post-tDCS (see also Tables 1 and 2). Table 8 summarizes the results of the multilevel model. While accuracy did not differ between the two groups (z = −0.69, p = .49), the cathodal group was reliably slower in the I condition than in the C condition (interaction: t = −2.00, p = .046). Neither the main effect of stimulation on RT's, nor the interaction between stimulation and condition on accuracy reached significance. As before, there was an advantage for the C trials in both accuracy (z = −7.57, p = <.001) and RT (t = 16.87, p = <.001). Together, the combination of close accuracy and slower RTs in the I condition point to a mild inhibitory effect in the cathodal condition on inhibiting the flankers.

**Table 8:** Summary of the fixed and random effects in the multi-level model in Experiment 3.

Accuracy					
	Fixed Effects	Coefficient	SE	z	Pr(>|z|)
	Intercept	2.2192	0.16134	13.755	<0.001
	Condition	−0.58119	0.07678	−7.569	<0.001
	Stimulation	−0.16384	0.23832	−0.687	0.492
	Condition*Stimulation	−0.12244	0.11003	−1.113	0.266
	**Random Effects**	**Variance**			
	Subject intercept	0.27901			

RT					
	Fixed Effects	Coefficient	SE	t	Pr(>|t|)
	Intercept	539.495	10.782	50.03	<0.001
	Condition	45.032	2.67	16.87	<0.001
	Stimulation	−19.096	15.994	−1.19	0.2325
	Condition*Stimulation	−7.992	3.997	−2	0.0456
	**Random Effects**	**Variance**			
	Subject intercept	1369.6			

Stimulation has two levels (cathodal and sham), and Condition has two levels (C and I). See text for details. * signifies an interaction.

To formally examine the flip in the results between experiments 2 and 3, both of which use similar stimulation timeline (but different tasks during the stimulation), we employed two models, one with accuracy and the other with RT as their dependent variables, and tested experiments 2a and 2b separately against Experiment 3. Each model contained Experiment, Condition, Stimulation and their interaction terms as its independent variables.

In the model comparing Experiment 2a against Experiment 3, there was a marginal interaction between Stimulation and Experiment in RT's (t = −1.88, p = 0.060). Other interactions were not significant (Table 9). In the model comparing Experiment 2b against Experiment 3, there was a significant three-way interaction between Stimulation, Condition and Experiment in RT's (t = −2.7, p = 0.007). Other interactions were not significant (Table 10). These analyses point to a difference between the effects of cathodal stimulation in Experiment 2a, b vs. Experiment 3.

**Table 9:** Summary of the fixed and random effects in the multi-level model testing the effect in Experiment 2a vs. Experiment 3.

Accuracy				
Fixed Effects	Coefficient	SE	z	Pr(>|z|)
Intercept	2.61549	0.2003	13.058	<0.001
Condition	−0.62145	0.10154	−6.12	<0.001
Stimulation	−0.65034	0.25428	−2.558	0.0105
Experiment	−0.39412	0.26308	−1.498	0.1341
Condition*Stimulation	−0.04805	0.1204	−0.399	0.6898
Condition*Experiment	0.03993	0.12732	0.314	0.7538
Stimulation*Experiment	0.48512	0.35802	1.355	0.1754
Condition*Stimulation*Experiment	−0.07432	0.16312	−0.456	0.6487
**Random Effects**	**Variance**			
Subject intercept	0.31562			

RT				
Fixed Effects	Coefficient	SE	t	Pr(>|t|)
Intercept	510.33316	13.14674	38.82	<0.001
Condition	49.18332	2.97729	16.52	<0.001
Stimulation	25.84272	16.85492	1.53	0.1252
Experiment	29.16439	17.39419	1.68	0.0936
Condition*Stimulation	−0.04362	3.90111	−0.01	0.9911
Condition*Experiment	−4.14913	3.98348	−1.04	0.2976
Stimulation*Experiment	−44.94144	23.86411	−1.88	0.0597
Condition*Stimulation*Experiment	−7.94891	5.55978	−1.43	0.1528
**Random Effects**	**Variance**			
Subject intercept	1531.9			

Stimulation has two levels (cathodal and sham), Condition has two levels (C and I), and Experiment has two levels (Exp 2a and Exp 3). See text for details. * signifies an interaction.

**Table 10:** Summary of the fixed and random effects in the multi-level model testing the effect in Experiment 2b vs. Experiment 3.

Accuracy				
Fixed Effects	Coefficient	SE	z	Pr(>|z|)
Intercept	2.06783	0.20056	10.31	<0.001
Condition	−0.42973	0.09274	−4.634	<0.001
Stimulation	−0.1041	0.25126	−0.414	0.6787
Experiment	0.15218	0.25964	0.586	0.5578
Condition*Stimulation	−0.23952	0.11307	−2.118	0.0341
Condition*Experiment	−0.1516	0.12041	−1.259	0.208
Stimulation*Experiment	−0.06026	0.34996	−0.172	0.8633
Condition*Stimulation*Experiment	0.11733	0.15777	0.744	0.4571
**Random Effects**	**Variance**			
Subject intercept	0.29289			

RT				
Fixed Effects	Coefficient	SE	t	Pr(>|t|)
Intercept	518.63	13.045	39.76	<0.001
Condition	41.59	3.262	12.75	<0.001
Stimulation	17.542	16.354	1.07	0.2835
Experiment	20.865	16.84	1.24	0.2154
Condition*Stimulation	7.548	4.135	1.83	0.068
Condition*Experiment	3.442	4.215	0.82	0.4141
Stimulation*Experiment	−36.638	22.738	−1.61	0.1071
Condition*Stimulation*Experiment	−15.54	5.75	−2.7	0.0069
**Random Effects**	**Variance**			
Subject intercept	1335.7			

Stimulation has two levels (cathodal and sham), Condition has two levels (C and I), and Experiment has two levels (Exp 2b and Exp 3). See text for details. * signifies an interaction.

## General Discussion

This paper was motivated by the diversity of behavioral results reported in studies where cathodal stimulation was employed to change performance in a cognitive task. At the behavioral level, reports of both better and worse (and sometimes no different than baseline) performance have appeared in the literature, making it difficult to understand the true effect of cathodal stimulation on behavior. This is particularly dangerous because strong *a priori* assumptions are often made in tDCS studies, where cathodal stimulation is *assumed* to induce inhibition.

We aimed to investigate the effect of two factors, often ignored in the literature, that we believe may have a direct bearing on the diversity of cathodal effects on behavior. These two factors were (a) whether testing is carried out during or after tDCS, and (b) in cases of post-tDCS testing, whether the activity the subject is performing during tDCS engages similar brain regions as the test task. It is not uncommon for researchers to examine post-tDCS cathodal effects on behavior [5], [6], [11], [13]–[16], [18], [21], [23]. Crucially, subjects' mental state during stimulation is often deemed unimportant in these reports, with some studies engaging subjects in the same task as the test task [5], [6], [13], [14], [18], some in another task or no task at all [11], [15], [16], [21], [23]. In three experiments, we studied the effect of cathodal stimulation on behavior when the experimental task was administered during (Experiment 1) vs. post tDCS (Experiments 2 and 3). When the task was administered post-tDCS, we manipulated whether subjects' cognitive activity during tDCS engaged the same cognitive/neural structures as the experimental task targeted by PFC stimulation (Experiment 3), or not (Experiment 2). Importantly, the experimental task was kept constant across all the main experiments, so any changes in behavior cannot be attributed to changes in the test task.

Before we summarize the results of the experiments, we review the predictions for the effects of stimulation in the letter Flanker task. Specifically, two types of effects can be expected: the main effect of stimulation, and the interaction between stimulation and condition (C vs. I), as the letter version of the Flanker task used in this study poses two types of demand on the PFC: (a) The classic Flanker effect, which is defined as the greater difficulty in responding to the incongruent condition (i.e. where the central letter is different from its flanking letters), compared to the congruent condition. If cathodal tDCS changes the selective processing of the target by inhibiting its flankers, this is expected to manifest as an interaction between stimulation and condition. (b) A demand on working memory. Four mapping variants must be learned, and this is made more difficult by the fact that four stimuli map onto only two responses, thus creating interference. Intertrial interval is unpredictable (i.e. less room for a planned rehearsal of mapping options), and a very quick response is required on each trial, where mapping must be retrieved from memory. This poses a serious demand on the PFC, regardless of whether the stimulus is congruent or incongruent. If cathodal tDCS alters working memory, we expect this to manifest as a main effect on both types of trials. Given the low spatial resolution of tDCS and mapping of both these functions to the lateral PFC, we expect to see traces of both of them in our experiments. Inspection of the pattern of results in Figures 1–3 shows that this is in fact the case, although the reliability of the two effects differs between the experiments.

Table 11 summarizes the results of the three experiments. The first candidate factor was timing of testing in relation to stimulation (during or post). The table shows that this factor alone cannot account for the variability in cathodal effects: participants were tested post-tDCS in both Experiments 2 and 3, but Experiment 2 yielded better performance compared to baseline, and Experiment 3 worse performance. However, the other experimental variable, cognitive activity during tDCS, did have a systematic effect on the results. Table 11 shows that when cathodal stimulation is applied to PFC while participants engage in a cognitive activity which poses considerable executive demands, as in Flanker and our object name reversal task, negative effects of stimulation on behavior emerge (Experiments 1 and 3). However, when the cognitive activity does not pose such demands, and is therefore unlikely to heavily engage the neural tissue under stimulation, the behavioral results are different (Experiment 2).

**Table 11:** Summary of the results of Experiments 1–3. “Timing” and “Task at the time of stimulation” denote the two experimental factors, and the last column shows the outcome.

Exp	Montage	Timing	Activity at the time of stimulation	Effect
1	F7 – mastoid	During	Letter Flanker (executive demand:^ high^)	Performance↓
**2**a,b	F7 – mastoid/supraorbital	Post	Simple categorization (executive demand: minimal)	Performance↑
**3**	F7 – mastoid	Post	Object name reversal (executive demand:^ high^)	Performance↓

The table shows that the outcome can be predicted from the task, but not from the timing manipulation. ↓  =  decline; ↑  =  improvement.

Does timing of the stimulation in relation to the task have any effects? Table 12 presents the results of an analysis comparing Experiment 1 to Experiment 3. As can be seen, none of the interaction terms between Experiment and Stimulation are significant. However, note that although in both experiments 1 and 3 we found evidence for facilitation, the effects showed up in different aspects of the task. In Experiment 1, behavioral facilitation manifested as a reliable decrease in the RT's (on average 30 ms; 95% CI = 2.5–60 ms), while this decrease was less dramatic, and not reliable, in Experiment 3 (on average 19 ms; 95% CI = −11–51 ms). On the other hand, Experiment 3 showed reliable facilitation for processing of the incongruent trials, which was absent in Experiment 1. This difference is not unexpected given that the Word-pair task preceding the Flanker task in Experiment 3 is heavily geared towards conflict processing, which participants in Experiment 1 do not have practice with. However, the difference in the locus of the effects limits our ability to draw definite conclusions from comparing experiments 1 and 3.

**Table 12:** Summary of the fixed and random effects in the multi-level model testing the effect in Experiment 1 vs. Experiment 3.

Accuracy				
Fixed Effects	Coefficient	SE	z	Pr(>|z|)
Intercept	2.16068	0.18335	11.785	<0.001
Condition	−0.51548	0.09351	−5.512	<0.001
Stimulation	0.20571	0.2604	0.79	0.43
Experiment	0.05531	0.23698	0.233	0.815
Condition*Stimulation	0.15451	0.14099	1.096	0.273
Condition*Experiment	−0.06515	0.12098	−0.539	0.59
Stimulation*Experiment	−0.36742	0.34197	−1.074	0.283
Condition*Stimulation*Experiment	−0.27713	0.17882	−1.55	0.121
**Random Effects**	**Variance**			
Subject intercept	0.2373			

RT				
Fixed Effects	Coefficient	SE	t	Pr(>|t|)
Intercept	548.795	11.901	46.11	<0.001
Condition	39.539	3.211	12.31	<0.001
Stimulation	−30.533	16.828	−1.81	0.0696
Experiment	−9.305	15.364	−0.61	0.5448
Condition*Stimulation	−2.727	4.483	−0.61	0.5429
Condition*Experiment	5.49	4.147	1.32	0.1856
Stimulation*Experiment	11.44	22.157	0.52	0.6056
Condition*Stimulation*Experiment	−5.264	5.961	−0.88	0.3771
**Random Effects**	**Variance**			
Subject intercept	1108.6			

Stimulation has two levels (cathodal and sham), Condition has two levels (C and I), and Experiment has two levels (Exp 1 and Exp 3). See text for details. * signifies an interaction.

Although the behavioral nature of our experiments does not allow for drawing a strong conclusion about the exact nature of the neural changes contributing to the change in the direction of the results, our findings point to the possibility that the effect of tDCS is dependent on the state of neuronal activation. In agreement with this, Vanneste et al. [47] reported differential responsiveness to tDCS as a function of different resting states; individuals with tinnitus were more likely to respond positively to bifrontal tDCS if their resting brain state showed higher gamma band activity in right primary and secondary auditory cortex, targeted by tDCS. State-dependent effects have been known for years, in the form of priming [48], and adaptation (where response to a sensory stimulus changes as a function of prolonged exposure to that stimulus [49]). Although there is disagreement about the exact underlying mechanisms of adaptation and priming [50], behavioral and neuroimaging studies have provided unequivocal evidence for such state-dependent effects.

Similar results have been obtained from the Transcranial Magnetic Stimulation (TMS) studies: When applied during a cognitive process, TMS generally induces inhibition (e.g., [51]), whereas facilitatory effects have been obtained with applications of single-pulse TMS shortly before the task [52], [53]. State-dependency explained this flip, and led to the revision of the role of repetitive TMS (rTMS). Originally, rTMS applied to the M1was considered to have inhibitory effects if its frequency was low (1 Hz), as evidenced by attenuated corticospinal excitability [54]–[56], while rTMS with higher frequency (≥5 Hz) was considered to have excitatory effects [57]. Studies of Lang et al. [58] and Siebner et al. [59] challenged this assumption by showing that the effects of TMS depended on the state of neuronal activation at the time of stimulation. Lang et al. [58] showed that preconditioning the neural tissue with cathodal tDCS before applying 5-Hz rTMS resulted in increased corticospinal excitability above the level of baseline, while preconditioning the tissue with anodal tDCS had the opposite effect. Siebner et al. [59] found a very similar pattern with 1-Hz rTMS, thus suggesting that the main factor in determining the outcome of stimulation was not the frequency of TMS, but the activation state of the neuronal tissue under stimulation. Similarly, Silvanto et al. [60] demonstrated a flip in the effects of TMS, when V5/MT was preconditioned with 1-Hz rTMS, prior to the application of online TMS. Without preconditioning, application of either online TMS, or 1-HZ rTMS to this region impaired performance on a simple motion detection task. However, when preconditioned first with the 1-Hz rTMS, application of online TMS caused an *improvement* in motion detection [60].

For the reasons discussed in the introduction, it is much easier to study the effects of stimulation (TMS or tDCS) in the motor as opposed to the cognitive domain, as the latter often draws on multiple parallel operations in different cortical regions and has much higher variability among individuals. The common engagement of multiple brain regions in cognitive tasks makes it even more susceptible to state-dependent effects. Bestmann et al. [61] had participants either rest their left hand or perform an isometric grip with that hand in the fMRI scanner, while receiving short pulse trains of TMS to the *left* dorsal premotor cortex (note that this is the ipsilateral hemisphere, which should not be directly involved in the motor grip of the left hand). Of interest, was the modulation of the BOLD response in the *right* motor areas. They demonstrated that during the left hand grip, TMS led to increased activity in the right motor areas, while it decreased the BOLD response when participants were resting their hand. These results suggest that state-dependent effects of stimulation can manifest in regions not directly under stimulation.

In spite of these challenges, we have shown that certain fundamental principles may apply to both motor and cognitive studies when stimulation is concerned; specifically, stimulation effects cannot, and should not, be viewed in isolation from the task at the time of stimulation. Instead, the outcome of stimulation must be viewed as an interaction between the stimulation and the activity of the tissue undergoing stimulation [62]. We acknowledge that this conclusion is based on behavioral evidence, and hope that these results will motivate further investigation of the nature the changes in the biological tissue undergoing tDCS, using electrophysiological measurements [63]. For now, we propose that our conclusion has direct implications for tDCS designs: For the most straightforward interpretation, effects must be studied during stimulation. If the test is administered post-tDCS, subjects' cognitive activity *during* stimulation must be carefully considered, and results must be interpreted with this crucial variable in mind.

## Supporting Information

Appendix S1 **The stimulus-incongruent condition.**
(DOCX)
