# Supplementary material for: Consequences of Cathodal Stimulation for Behavior: When Does It Help and When Does It Hurt Performance?
Source: PLoS One. 2014 Jan 7;9(1):e84338. doi: 10.1371/journal.pone.0084338 (PMC3883650; doi:10.1371/journal.pone.0084338)
Supplement: Appendix S1 — The stimulus-incongruent condition. (DOCX) [file pone.0084338.s001.docx]

The data in the stimulus incongruent (SI) condition were not included in the main analyses, because of the indeterminacy associated with the process underlying a correct response. In a sequence such as SSXSS (where both S and X map onto the same response), a “correct response” is obtained when the participants respond to the central letter, as well as when they actually fail, and respond by mistake to the flanking letters. This failure which manifests as a correct response is behaviorally indistinguishable from successfully inhibiting flankers, and inclusion of such trials only adds noise to the analyses. However, our results replicated previous findings of Van Venn and Carter (2002), who demonstrated that RT’s for the SI trials fall in between the congruent and incongruent trials. Below is a detailed report of accuracy (panel a) and reaction times (panel b) in the SI condition for all the experiments.

**(a)**

|  | **Cathodal** | | | | **Sham** | | | |
| --- | --- | --- | --- | --- | --- | --- | --- | --- |
|  | **Congruent** | | **Incongruent** | | **Congruent** | | **Incongruent** | |
|  | **Mean** | **SE** | **Mean** | **SE** | **Mean** | **SE** | **Mean** | **SE** |
| **Exp 1** | 0.89 | 0.025 | 0.82 | 0.028 | 0.91 | 0.010 | 0.88 | 0.10 |
| **Exp 2a** | 0.92 | 0.013 | 0.87 | 0.018 | 0.86 | 0.021 | 0.77 | 0.027 |
| **Exp 2b** | 0.88 | 0.018 | 0.83 | 0.020 | 0.86 | 0.021 | 0.77 | 0.027 |
| **Exp 3** | 0.89 | 0.020 | 0.82 | 0.028 | 0.88 | 0.016 | 0.79 | 0.026 |

**(b)**

|  | **Cathodal** | | | | **Sham** | | | |
| --- | --- | --- | --- | --- | --- | --- | --- | --- |
|  | **Congruent** | | **Incongruent** | | **Congruent** | | **Incongruent** | |
|  | **Mean** | **SE** | **Mean** | **SE** | **Mean** | **SE** | **Mean** | **SE** |
| **Exp 1** | 547.8 | 11.2 | 587.3 | 10.7 | 517.8 | 9.17 | 555.2 | 7.68 |
| **Exp 2a** | 509.8 | 14.2 | 558.7 | 14.7 | 534.1 | 11.5 | 582.2 | 10.9 |
| **Exp 2b** | 518.4 | 9.26 | 560.3 | 9.74 | 534.1 | 11.5 | 582.2 | 10.9 |
| **Exp 3** | 537.8 | 11.8 | 581.7 | 11.03 | 520.1 | 11.2 | 556.4 | 10.3 |
